# Comparison of genetic variations between high- and low-risk *Listeria monocytogenes* isolates using whole-genome *de novo* sequencing

**DOI:** 10.3389/fmicb.2023.1163841

**Published:** 2023-07-18

**Authors:** Jihye Ryu, Yukyung Choi, Yohan Yoon

**Affiliations:** ^1^Department of Food and Nutrition, Sookmyung Women’s University, Seoul, Republic of Korea; ^2^Risk Analysis Research Center, Sookmyung Women’s University, Seoul, Republic of Korea

**Keywords:** enoki mushroom, smoked duck, processed ground meat product, next-generation sequencing, genetic variation, *Listeria monocytogenes*

## Abstract

In this study, genetic variations and characteristics of *Listeria monocytogenes* isolates from enoki mushrooms (23), smoked ducks (7), and processed ground meat products (30) were examined with respect to hemolysis, virulence genes, growth patterns, and heat resistance. The isolates that showed the highest pathogenicity and the lowest pathogenicity were analyzed to obtain the whole-genome sequence, and the sequences were further analyzed to identify genetic variations in virulence, low-temperature growth-related, and heat resistance-related factors. All isolates had *β*-hemolysis and virulence genes (*act*A, *hly*A, *inl*A, *inl*B, and *plc*B). At low temperatures, isolates with high growth (*L. monocytogenes* strains SMFM 201803 SD 1-1, SMFM 201803 SD 4-2, and SMFM 201804 SD 5-3) and low growth (*L. monocytogenes* strains SMFM 2019-FV43, SMFM 2019-FV42, and SMFM 2020-BT30) were selected. Among them, *L. monocytogenes* SMFM 201804 SD 5-3 showed the highest resistance at 60°C and 70°C. The strains SMFM 201804 SD 5-3 (high-risk) and SMFM 2019-FV43 (low-risk) harbored 45 virulence genes; 41 single nucleotide variants (SNVs) were identified between these two isolates. A comparison of 26 genes related to low-temperature growth revealed 18 SNVs between these two isolates; a comparison of the 21 genes related to heat resistance revealed 16 SNVs. These results indicate that the differences in the pathogenicity of *L. monocytogenes* SMFM 201804 SD 5-3 and *L. monocytogenes* SMFM 2019-FV43 are associated with the SNVs identified in virulence genes, low-temperature growth-related genes, and heat resistance-related genes.

## 1. Introduction

*Listeria monocytogenes* contaminates various foods, such as meat, fresh agricultural products, and smoked salmon; thus, foodborne illnesses caused by *L. monocytogenes* are frequent, throughout the year (Sheng et al., 2018). In Korea, recalls of processed meat products due to *L. monocytogenes* contamination occur continuously (Lee and Yoon, 2021). *L. monocytogenes* is a gram-positive facultative anaerobe that causes listeriosis in humans (Chen and Knabel, 2007; Yang and Moon, 2021). The pathogen may also cause severe infectious diseases in pregnant women and the immunocompromised (Shin et al., 2007). The fatality rate of individuals infected with *L. monocytogenes* is 20–30%; this pathogen may cause infection even when present in low numbers compared to other pathogenic bacteria (Lee, 2010). The minimum infectious dose varies among patients and foods, but it is generally estimated 10^2^–10^3^ CFU/g (Park et al., 2014). *L. monocytogenes* can grow under anaerobic conditions, and thus, they can grow under vacuum or nitrogen-filled packaging (Duffy et al., 1994). It can grow even at refrigerated temperatures and survive under conditions of high-salt (≥10%), extreme acidity, and carbon-source depletion (Liu et al., 2006). Also, *L. monocytogenes* is one of the most heat-resistant pathogens and poses a notable risk to food safety, particularly when mild heat is treated in food processing and preparation (Pöntinen et al., 2017). These survival and viability characteristics of *L. monocytogenes* raise concerns regarding foodborne illnesses caused by the consumption of foods contaminated with this pathogen.

The advancement of next-generation sequencing (NGS) techniques has allowed the cost- and time-effective sequencing of DNA for microbial genome research. NGS techniques are used in various fields, including verification of the functionality of probiotics and investigation of the causes of foodborne outbreaks. They have also been used to establish a database of genomic information for foodborne pathogenic bacteria and to analyze the safety of microorganisms (Kwon et al., 2019; Cao et al., 2021). Whole-genome sequencing (WGS)-based methods have contributed to the identification of outbreak sources in several listeriosis outbreaks (Lüth et al., 2018). According to Hilliard et al. (2018), WGS analysis is a valuable methodology for classifying *L. monocytogenes* isolates and identifying virulence islands that may influence infectivity. WGS is increasingly used in the United States to facilitate detecting, investigating, and controlling foodborne bacterial outbreaks (Brown et al., 2019). In Ireland, WGS and phenotypic assays were used to explain the virulence of *L. monocytogenes* isolates (Stratakos et al., 2020). WGS provides an opportunity to determine strain characteristics typically obtained through resource-intensive traditional methodologies such as species identification, serotyping, virulence, and antimicrobial resistance profiling, all of which can be consolidated into a single WGS workflow (Ribot et al., 2019). The objective of this study was to identify phenotypes and genetic variations among *L. monocytogenes* isolates using WGS.

## 2. Materials and methods

### 2.1. Isolation of *Listeria monocytogenes*

The *L. monocytogenes* isolates from enoki mushrooms and other isolates (7 isolates from smoked ducks and 30 isolates from processed ground meat products) previously studied by Park et al. (2021) were used in the present study.

A total of 127 enoki mushrooms were collected from supermarkets in Korea between August 2019 and March 2020. The samples were transported in a cooler to our laboratory. Briefly, 25 g of each sample was placed in sample bags (3 M™, St. Paul, MN, USA) containing 225 mL of Listeria Enrichment Broth (Becton Dickinson and Company). The sample bags were hand-shaken for 1 min and incubated at 30°C for 48 h to enrich *L. monocytogenes*.

One-milliliter aliquots of the enriched cultures were spread-plated on Chrom agar (CHROM™ agar Listeria; Paris, France), and the plates were incubated at 37°C for 24 h. The isolated colonies were subjected to real-time PCR analysis to investigate the presence of the *iap* gene for the confirmatory test of *L. monocytogenes* [Food and Drug Administration (FDA), 2018] with a 264-bp DNA fragment, using suitable primers (F: 5′-TGG GAT TGC GGT AAC AGC AT-3′ R: 5′-TA TCA ACA CCA GCG CCA CT-3′) (Kim, 2019). A single colony was suspended in 50 μL of 0.25% sodium dodecyl sulfate (Biosesang, Gyeonggi-do, Korea)–0.05 N NaOH solution (Daejung, Gyeonggi-do, Korea) and 100 μL of dH_2_O. The suspensions were heated at 99°C for 15 min, left at room temperature (25°C) for 3 min, and centrifuged at 5,989 × *g* at 4°C for 3 min; the supernatants were used as DNA templates. Real-time PCR amplification was performed using a Rotor-Gene SYBR^®^ Green PCR Kit (Qiagen, Hilden, Germany). Briefly, 1 μL DNA template was mixed with 2.5 μL of forward primer, 2.5 μL of reverse primer, 12.5 μL of SYBR green master mix, and 6.5 μL of dH_2_O. For *iap* amplification, an initial denaturation step at 95°C for 90 s and 35 cycles of denaturation at 95°C for 5 s, annealing at 56°C for 10 s, and extension at 72°C for 5 s were conducted. Finally, a 5-min extension was performed at 72°C. Amplified PCR products were loaded onto a 1.5% agarose gel and visualized under UV light.

### 2.2. Analysis of hemolytic property

Isolated colonies of *L. monocytogenes* were inoculated in 10 mL tryptic soy broth with 0.6% yeast extract (TSBYE; Becton Dickinson and Company) and incubated at 30°C overnight. Then, 100-μL aliquots of the cultures were transferred to fresh 10 mL TSBYE and incubated at 30°C for another 24 h. The sub-cultures were streaked onto Columbia agar with 5% sheep blood (bioMérieux, Mercy 1′Etoile, France) and incubated at 30°C for 48 h. Hemolytic properties (*α*-, *β*-, or *γ*-hemolysis) were determined by observing the zones formed around the bacterial colonies. *β*-hemolysis (complete hemolysis) was confirmed if a clear zone around the bacteria was observed.

### 2.3. Detection of virulence genes

Isolated colonies of *L. monocytogenes* isolates were suspended in 50 μL of 0.25% sodium dodecyl sulfate–0.05 N NaOH solution. One hundred microliters of sterile dH_2_O was added to the suspension, and the mixtures were incubated at 99°C for 15 min. Aliquots (2 μL) of the mixtures were mixed with the components of the Phire Hot Start II DNA Polymerase Kit (Thermo Fisher Scientific, Waltham, MA, US), Taq DNA polymerase mix (20 mM Tris–HCl; pH 7.4 at 25°C, 0.1 mM EDTA, 1 mM DTT, 100 mM KCl, 200 μg/mL BSA, and 50% glycerol), 1X reaction buffer (1.5 mM MgCl_2_), 200 μM deoxynucleoside triphosphates (dNTPs), and 0.5 μM of each of the virulence gene primers. Five virulence genes (*act*A, *hly*A, *inl*A, *inl*B, and *plc*B) were detected with PCR using the primers listed in Table 1. PCR was performed on a Rotor-Gene Q thermal cycler (Qiagen) at the following conditions: initial denaturation at 98°C for 30 s, followed by 35 cycles of 98°C for 5 s, 60°C for 5 s, and 72°C for 10 s, with a final extension step at 72°C for 1 min. Amplified PCR products were loaded onto a 1.5% agarose gel and visualized under UV light.

**Table 1:** Information of primers used for the detection of virulence genes (*act*A, *hly*A, *inl*A, *inl*B, and *plc*B) in *Listeria monocytogenes*.

Target gene	Primer sequence (5′ to 3′)		Size (bp)	Reference
*act*A	Forward	GAC GAA AAT CCC GAA GTG AA	268, 385	Gianfranceschi et al. (1998)
	Reverse	CTA GCG AAG GTG CTG TTT CC		
*hly*A	Forward	GCA TCT GCA TTC AAT AAA GA	174	Riedo et al. (1994)
	Reverse	TGT CAC TGC ATC TCC GTC GT		
*inl*A	Forward	CCT AGC AGG TCT AAC CGC AC	255	Khelef et al. (2006)
	Reverse	TCG CTA ATT TGG TTA TGC CC		
*inl*B	Forward	AAA GCA CGA TTT CAT GGG AG	146	Jung et al. (2003)
	Reverse	ACA TAG CCT TCT TTG GTC GGG		
*plc*B	Forward	GGG AAA TTT GAC ACA GCG TT	261	Winters et al. (1999)
	Reverse	ATT TTC GGG TAG TCC GCT TT		

### 2.4. Comparing the growth patterns of *Listeria monocytogenes* isolates cultured at low temperature

As described in the section “2.2. Analysis of hemolytic property,” *L. monocytogenes* isolates were cultured and then sub-cultured in TSBYE at 30°C overnight. The sub-cultures were centrifuged at 1,912 × *g* and 4°C for 15 min. Cell pellets were washed twice with phosphate-buffered saline (PBS), and the suspensions were diluted with PBS and inoculated at 5 Log CFU/mL. The aliquots (0.2 mL) of the inocula of each *L. monocytogenes* isolates were inoculated into tubes containing 20 mL of TSBYE and incubated at 4°C for 288 h. During incubation, the cultures of *L. monocytogenes* were diluted with buffered peptone water (BPW; Bacto, Becton, Dickinson, Sparks, MD, USA), and 0.1-mL aliquots of the diluents were plated on tryptic soy agar with 0.6% yeast extract (TSAYE; Becton Dickinson and Company). The plates were incubated at 30°C for 48 h, and the colonies on the plates were manually counted. The six for each strain were repeated for each time point.

### 2.5. Comparing the growth patterns of *Listeria monocytogenes* isolates after vacuum treatment

Aliquots (0.1 mL) of the inocula were placed on a 10-g beef rump. The inoculated samples were vacuum-packed and stored at 4°C for 20 days. The samples were analyzed every 5 days. The samples were aseptically removed from the vacuum bags and placed in filter bags (3 M™) containing 20 mL BPW. The samples were then pummeled for 1 min in a pummeler (BagMixer; Interscience, St. Nom, France). Aliquots (1 mL) of the homogenates were diluted with BPW, and 0.1 mL of the diluents was spread-plated on Palcam agar (Becton Dickinson and Company). The plates were incubated at 30°C for 48 h, and the colonies on the plates were manually counted. The six for each strain were repeated for each time point.

### 2.6. Heat resistance

Aliquots (0.1 mL) of the inoculum were inoculated into tubes containing 9.9 mL of TSBYE; the tubes were preheated at 60°C and 70°C in a water bath. After 0, 3, 5, 8, and 10 min at 60°C and 0, 0.17, 3, 5, 8, and 10 min at 70°C, one-milliliter aliquots of the samples were retrieved and diluted with BPW, and 0.1-mL aliquots of the diluents were spread-plated on TSAYE, respectively. The plates were incubated at 30°C for 48 h, and the colonies on the plates were manually counted. The cell counts were then used to calculate *D*-values (decimal reduction time) as follows:

$$D_{T}=t/\left(Log\;N_{0}-Log\;\mathrm{Nt}\right),$$

Where *T* is temperature, *t* is time, Log N_0_ is the initial number of bacteria, and Log Nt is the number of bacteria remaining at time. The six for each strain were repeated for each time point.

### 2.7. Whole-genome sequencing

#### 2.7.1. DNA extraction and library preparation

After sub-culturing as described in the section “2.2. Analysis of hemolytic property” 3-mL aliquots of the sub-cultures were precipitated at 5,264  × *g* for 30 s. The cell pellets were used for extraction of DNA with the DNeasy Blood and Tissue Kit (Qiagen), according to the manufacturer’s protocol. Briefly, 5 μg of each extracted DNA sample was used to construct a library using the SMRTbell™ Template Prep Kit 1.0 (PN 100-259-100) (Pacific Biosciences, Menlo Park, CA, USA) following the manufacturer’s instructions. Fragments smaller than 20 kb of the SMRTbell template were removed using the BluePippin size selection system (Sage Science, Beverly, MA, USA) to construct large-insert libraries. The constructed library was validated using the 2100 Bioanalyzer (Agilent, Santa Clara, CA, USA) for quality control.

#### 2.7.2. Sequencing and *de novo* assembly

The SMRTbell libraries were annealed to sequencing primers, and DNA polymerase was bound to the complex using the DNA/Polymerase Binding Kit P6 (Pacific Biosciences). This polymerase-SMRTbell-adaptor complex was loaded into SMRT cells (Pacific Biosciences) and sequenced using the PacBio RS II sequencing platform (Pacific Biosciences) which produce continuous long read (CLR). Long contigs were generated via *de novo* assembly (DNA LinkInc., Seoul, South Korea), and gene annotation and prediction were performed to analyze their genetic properties.

#### 2.7.3. Analysis of genetic factors and genetic variations

Genetic characteristics of two high-risk and low-risk *L. monocytogenes* isolates were analyzed for 45 virulence factors, 26 low-temperature growth-related factors, and 21 heat resistance-related factors using CLC Genomics Workbench ver. 12.0 (Qiagen) (Table 2). The sequences of these factors were obtained from the NCBI GenBank database. The presence of genetic factors in the two *L. monocytogenes* isolates was determined using a Basic Local Alignment Search Tool (BLAST), and genetic variations were assessed by comparing the sequences of each gene.

**Table 2:** The list of genetic factors of *Listeria monocytogenes.*

Genetic factor	Function	Gene
Virulence factor	Adherence	*dlt*A, *fbp*A, *ami*, *inl*F*, inl*J, *lap, lap*B
	Enzyme	*mpl*, *plc*B, *plc*A, *stp*
	Immune modulator	*inl*C, *inl*K, *lnt*A
	Intracellular survival	*lpl*A1, *opp*A, *prs*A2, *hpt*
	Invasion	*aut*, *iap/cwh*A, *gtc*A, *inl*A, *inl*B, *inl*P*, lpe*A*, vip*
	Iron uptake	*hbp*2
	Nucleation-promoting factor	*act*A
	Bile resistance	*bsh*
	Peptidoglycan modification	*oat*A, *pdg*A
	Regulation	*agr*A, *agr*C, *che*A, *che*Y, *lis*R*, lis*K, *prf*A, *vir*R, *vir*S
	Surface protein anchoring	*lgt, lsp*A, *srt*A, *srt*B
	Toxin	*hly*
Low-temperature growth-related factor	Cell-membrane-associated protein genes	*opp*A, *gbu*A, *gbu*B, *gbu*C, *fla*A, *bet*L, *fbp, mot*A, *flh*A
	Cold stress adaptive regulatory protein genes	*htp*, *deg*U, *yycJ, lhk*A
	Cold shock phase protein genes	*csp*LA, *fri, csp*D, *csp*B
	Other cold stress protein genes	*trx*B, trpG, *ltr*A, *ltr*B, *ltr*C
	General stress response protein genes	*gro*EL, *clp*P, *sig*B, *rsb*U
Heat resistance-related factor	Heat shock protein genes	*dna*J, *dna*K, *grp*E, *hrc*A, *gro*EL, *gro*ES, *lmo*0229, *lmo*2030, *lmo*0231, *lmo*1138, *lmo*1279, *clp*C, *clp*E, *clp*B, *clp*P, *hsl*O, *lmo*0942, *lmo*0963, *lmo*0056, *lmo*0222, *lmo*0292

### 2.8. Statistical analysis

Statistical analysis was performed using SAS^®^ version 9.4 (SAS Institute Inc., Cary, NC, USA). Significant differences among strains were determined using analysis of covariance and a general linear model at *α* = 0.05.

## 3. Results and discussion

### 3.1. Isolation and identification of *Listeria monocytogenes*

In this study, 23 *L. monocytogenes* isolates from 127 enoki mushrooms (Table 3), and 37 *L. monocytogenes* isolates (7 isolates from smoked ducks and 30 isolates from processed ground meat products) previously isolated (Table 4) were obtained. The *iap* gene for the confirmatory test of *L. monocytogenes* was detected in all 23 *L. monocytogenes* isolates from 127 enoki mushrooms (data not shown).

**Table 3:** Information regarding *Listeria monocytogenes* isolates from enoki mushrooms purchased from a market in Korea.

No.	*L. monocytogenes* isolate	Date of purchase
1	SMFM 2019-FV41	August 2019
2	SMFM 2019-FV42	August 2019
3	SMFM 2019-FV43	August 2019
4	SMFM 2019-FV44	August 2019
5	SMFM 2019-FV45	August 2019
6	SMFM 2019-FV46	August 2019
7	SMFM 2019-FV47	August 2019
8	SMFM 2019-FV48	September 2019
9	SMFM 2019-FV49	September 2019
10	SMFM 2019-FV50	September 2019
11	SMFM 2019-FV51	September 2019
12	SMFM 2019-FV52	September 2019
13	SMFM 2019-FV53	September 2019
14	SMFM 2019-FV54	September 2019
15	SMFM 2019-FV55	September 2019
16	SMFM 2019-FV56	September 2019
17	SMFM 2019-FV57	October 2019
18	SMFM 2020-FV1	March 2020
19	SMFM 2020-FV2	March 2020
20	SMFM 2020-FV3	March 2020
21	SMFM 2020-FV4	March 2020
22	SMFM 2020-FV5	March 2020
23	SMFM 2020-FV6	March 2020

**Table 4:** Information regarding *Listeria monocytogenes* isolates from smoked ducks and processed ground meat products.

Food	*L. monocytogenes* isolate	Date of purchase	Place of purchase
Smoked duck	SMFM 201803 SD 1-1	March 2018	Market
	SMFM 201803 SD 4-1		
	SMFM 201803 SD 4-2		
	SMFM 201804 SD 5-2	April 2018	
	SMFM 201804 SD 5-3		
	SMFM 201804 SD 6-2		
	SMFM 201804 SD 7-1		
Processed ground meat product	SMFM 2020-BT1	January 2020	Online market
	SMFM 2020-BT2		
	SMFM 2020-BT3		
	SMFM 2020-BT4		
	SMFM 2020-BT5		
	SMFM 2020-BT6		
	SMFM 2020-BT8		
	SMFM 2020-BT9		
	SMFM 2020-BT10		
	SMFM 2020-BT11		
	SMFM 2020-BT12		
	SMFM 2020-BT13		
	SMFM 2020-BT14		
	SMFM 2020-BT15		
	SMFM 2020-BT16		
	SMFM 2020-BT17		
	SMFM 2020-BT18		
	SMFM 2020-BT19		
	SMFM 2020-BT20		
	SMFM 2020-BT21		
	SMFM 2020-BT22		
	SMFM 2020-BT23		
	SMFM 2020-BT24		
	SMFM 2020-BT25		
	SMFM 2020-BT26	February 2020	
	SMFM 2020-BT27		
	SMFM 2020-BT28		
	SMFM 2020-BT29		
	SMFM 2020-BT30	April 2020	
	SMFM 2020-BT31		

### 3.2. Hemolytic property

**Figure 1:**
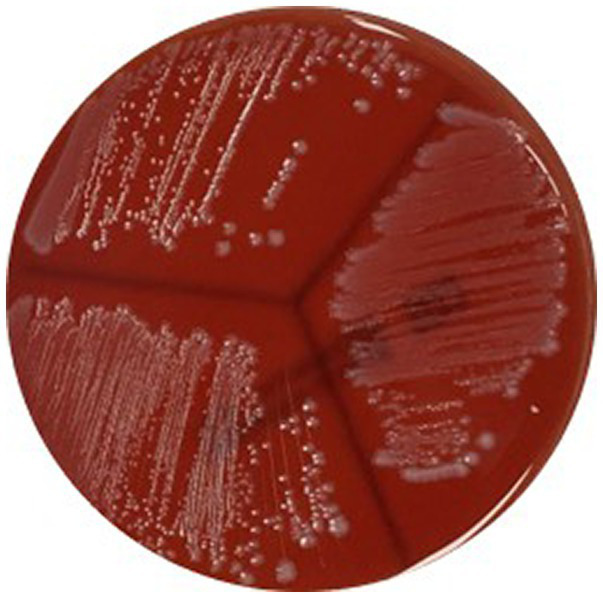
*β*-hemolytic property of *Listeria monocytogenes* SMFM 201803 SD 4-2, *Listeria monocytogenes* SMFM 201804 SD 5-2, and *Listeria monocytogenes* SMFM 201804 SD 6-2.

*Listeria* spp. which causes *β*-hemolysis is mostly pathogenic, and most of the isolated *L. monocytogenes* cause *β*-hemolysis (Farber and Peterkin, 1991). Examinations showed that all *L. monocytogenes* isolates caused *β*-hemolysis with narrow zone (Figure 1).

### 3.3. Virulence genes

**Figure 2:**
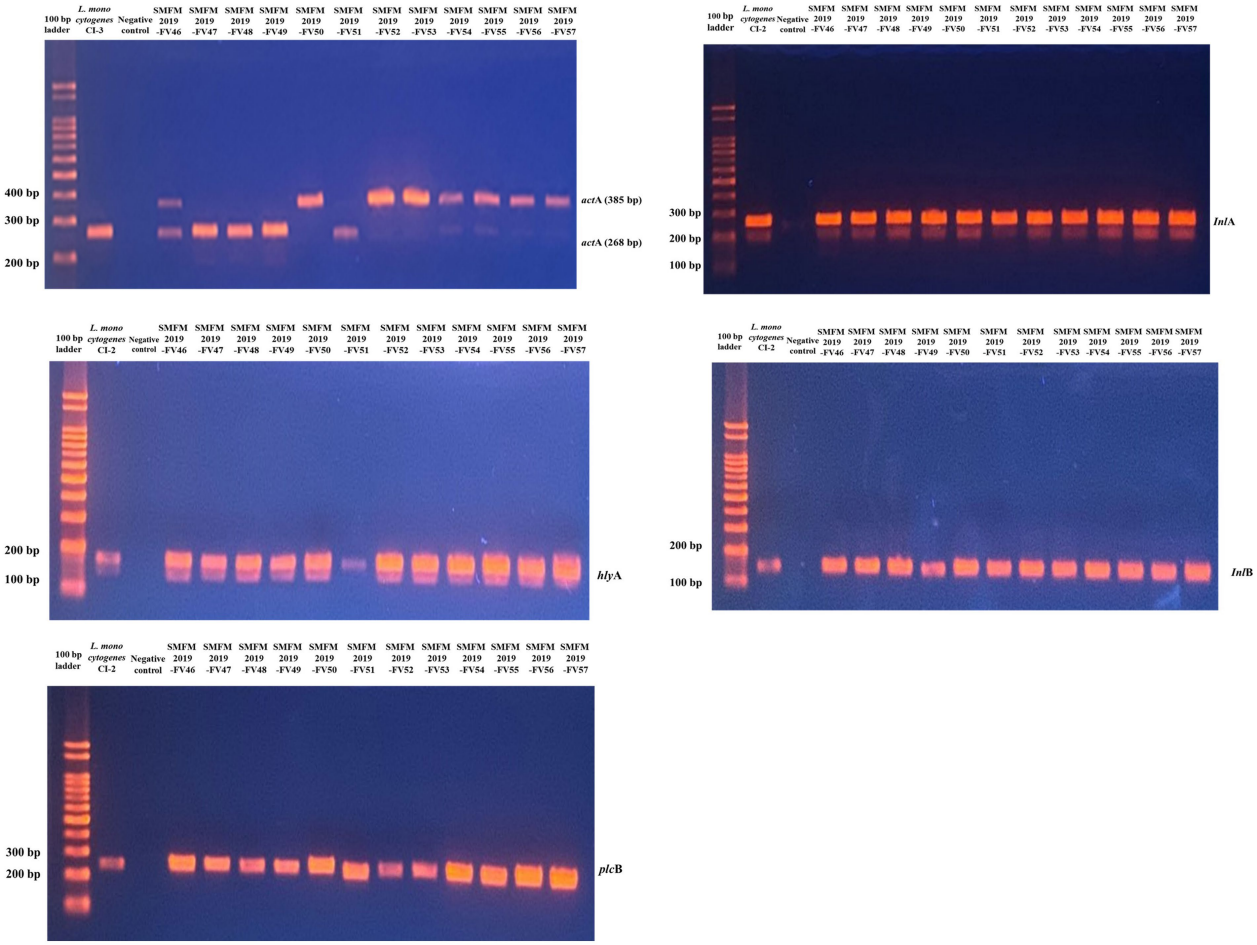
Gel image of PCR results for 12 *Listeria monocytogenes* isolates targeting *act*A, *hly*A, *inl*A, *inl*B, and *plc*B genes.

Five virulence genes (*act*A, *hly*A, *inl*A, *inl*B, and *plc*B) were detected in all *L. monocytogenes* isolates (Figure 2). In another study, most *L. monocytogenes* isolates harbored these virulence genes, and polymorphisms in the *act*A gene have been found between carcass and human isolates (Oh et al., 2018). *Act*A in *L. monocytogenes* encodes the surface protein *act*A, which is responsible for actin-based motility and cell-to-cell spread (Vàzquez-Boland et al., 2001). Rezaei et al. (2019) showed that the presence of *hly*A and *iap* in *L. monocytogenes* is effective in increasing its aggressiveness and pathogenicity. Cellular invasion requires the presence of bacterial surface proteins internalin A (*inl*A) and/or *inl*B and their interaction with the cellular receptors E-cadherin and/or Met, respectively (Kühbacher et al., 2018). Virulence-associated genes *plc*A and *plc*B encode phosphatidylinositol phospholipase C (PI-PLC) and phosphatidyl-choline phospholipase (PC-PLC), respectively. Both phospholipases are related to effective escape from the phagocytic vacuole into the cytoplasm (Gründling et al., 2003; Hadjilouka et al., 2016). These results indicate that the isolates examined in this study are pathogenic to humans.

### 3.4. Growth of *Listeria monocytogenes* isolates at 4°C

**Figure 3:**
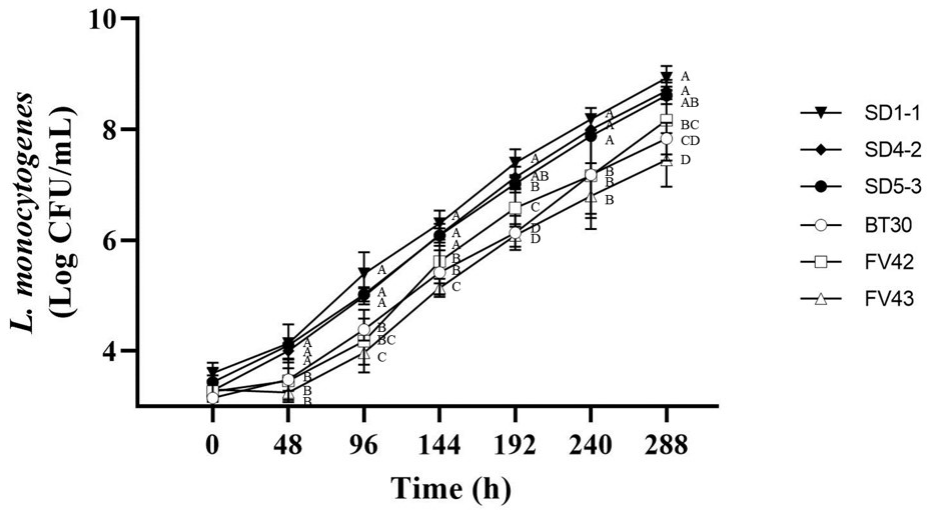
Cell counts of *Listeria monocytogenes* isolates in tryptic soy broth with 0.6% yeast extract during storage at 4°C. Error bars in the figure mean standard error. ^A–D^ means in the figure with different letters are significantly different (*p* < 0.05).

According to Kim et al. (1995), *L. monocytogenes* isolates from humans do not grow at 4°C; however, Seeliger and Jones (1986) reported that most *L. monocytogenes* could grow at 4°C. In this study, all the *L. monocytogenes* isolates showed growth at 4°C (Figure 3). Considering growth rates based on statistical difference over all time points (0, 48, 96, 144, 192, 240, and 288 h), high and low growth group were divided. *L. monocytogenes* SMFM 201803 SD 1-1 was in high growth group at all time points (48, 96, 144, 192, 240, and 288 h), and *L. monocytogenes* SMFM 201803 SD 4-2 was in high growth group at five time points (48, 144, 192, 240, and 288 h). *L. monocytogenes* SMFM 201804 SD 5-3 was in the high growth group at four points (48, 144, 240, and 288 h). *L. monocytogenes* SMFM 2019-FV43 was in the low growth group at all time points (48, 96, 144, 192, 240, and 288 h). *L. monocytogenes* SMFM 2020-BT30 was in low growth group at four time points (144, 192, 240, and 288 h), and *L. monocytogenes* SMFM 2019-FV42 was in low growth group at three time points (48, 96, and 240 h). Three (*L. monocytogenes* SMFM 201803 SD 1-1, *L. monocytogenes* SMFM 201803 SD 4-2, and *L. monocytogenes* SMFM 201804 SD 5-3) with high growth and three (*L. monocytogenes* SMFM 2019-FV42, *L. monocytogenes* SMFM 2019-FV43, and *L. monocytogenes* SMFM 2020-BT30) with low growth at 4°C were selected (Figure 3). The average cell counts (288 h – 0 h) of the high and low growth group were 5.3 and 4.6 Log CFU/mL, respectively. The three high-growth isolates were from smoked ducks; two low-growth isolates were from enoki mushrooms, while one low-growth isolate was from to the processed ground meat product.

### 3.5. Growth of *Listeria monocytogenes* isolates in vacuum

**Figure 4:**
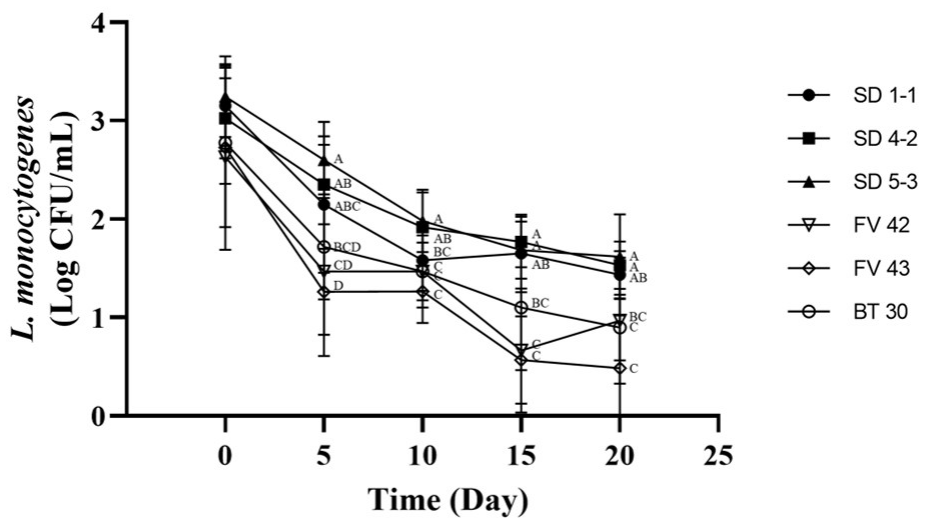
Cell counts of *Listeria monocytogenes* isolates inoculated in beef during storage under vacuum at 4°C. Error bars in the figure mean standard error. ^A–D^ means in the figure with different letters are significantly different (*p* < 0.05).

Isolates with high and low growth (three each) at 4°C were further analyzed to compare their growth under vacuum conditions at 4°C. Duffy et al. (1994) showed that *L. monocytogenes* can grow in vacuum-packed foods stored in a refrigerator. However, in our study, the *L. monocytogenes* isolates showed high mortality rates (Figure 4). Isolates with high growth at 4°C (*L. monocytogenes* SMFM 201803 SD 1-1, *L. monocytogenes* SMFM 201803 SD 4-2, and *L. monocytogenes* SMFM 201804 SD 5-3) had better survival under vacuum conditions than those with low growth (*L. monocytogenes* SMFM 2019-FV42, *L. monocytogenes* SMFM 2019-FV43, and *L. monocytogenes* SMFM 2020-BT30) (Figure 4). These results showed that high-growth *L. monocytogenes* isolates in low temperature survive long in vacuum conditions.

### 3.6. Heat resistance

The *D*_60_ and *D*_70_ values of the *L. monocytogenes* isolates, which describe heat resistance at 60°C and 70°C, ranged 2.37–3.55 min and 1.83–2.20 min, respectively (Table 5). According to Tangwatcharin et al. (2019), *D*-values of *L. monocytogenes* in inoculated restructured goat steaks ranged from 7.27 min at 60°C to 0.46 min at 70°C. In our study, at 60°C, *L. monocytogenes* SMFM 201804 SD 5-3 (3.55 min) had higher *D*_60_ value than the others followed by *L. monocytogenes* SMFM 2019-FV42 (3.17 min), and the *D*_70_ value (2.20 min) of *L. monocytogenes* SMFM 201804 SD 5-3 was also higher than that of the other isolates (Table 5). This indicated that *L. monocytogenes* SMFM 201804 SD 5-3 had the highest heat resistance.

**Table 5:** *D*-values of *Listeria monocytogenes* isolates at 60°C and 70°C.

*L. monocytogenes* isolate	*D*_60°C_ (min)	*D*_70°C_ (min)
ATCC13932	2.37^C^	1.90^BC^
SMFM 201803 SD 1-1	2.80^BC^	2.07^AB^
SMFM 201803 SD 4-2	2.47^C^	1.83^BC^
SMFM 201804 SD 5-3	3.55^A^	2.20^A^
SMFM 2019-FV42	3.17^AB^	1.85^C^
SMFM 2019-FV43	2.60^C^	1.97^BC^
SMFM 2020-BT30	2.65^C^	1.83^C^

^A–C^means in the same column with different letters are significantly different (*p* < 0.05).

There was association between cold and heat resistance. The growth of *L. monocytogenes* SMFM 201804 SD 5-3 at 4°C was significantly higher than that of *L. monocytogenes* SMFM 2020-BT30 and *L. monocytogenes* SMFM 2019-FV43 (*p* < 0.05, Figure 3). The *D*_60_ and *D*_70_ of *L. monocytogenes* SMFM 201804 SD 5-3 were significantly higher than those of *L. monocytogenes* SMFM 2019-FV43 (*p* < 0.05, Table 5). These results show a correlation between *L. monocytogenes* SMFM 201804 SD 5-3 (high cold- and heat-resistance) and *L. monocytogenes* SMFM 2019-FV43 (low cold- and heat-resistance) for growth at low temperature and survival in heat resistance. In the case of *L. monocytogenes* SMFM 201803 SD 1-1, the growth of the isolate was significantly higher than *L. monocytogenes* SMFM 2020-BT30, *L. monocytogenes* SMFM 2019-FV42, and *L. monocytogenes* SMFM 2019-FV43 at 4°C (*p* < 0.05, Figure 3), and there was no significant difference in *D*_60_. However, *D*_70_ of *L. monocytogenes* SMFM 201803 SD 1-1 was higher than that of *L. monocytogenes* SMFM 2019-FV42 and *L. monocytogenes* SMFM 2020-BT30 (*p* < 0.05, Table 5). *L. monocytogenes* SMFM 201803 SD 4-2 grew significantly higher than *L. monocytogenes* SMFM 2020-BT30, *L. monocytogenes* SMFM 2019-FV42, and *L. monocytogenes* SMFM 2019-FV43 at 4°C (*p* < 0.05, Figure 3), however, there was no significant difference in both *D*_60_ and *D*_70_.

### 3.7. Selection of high- and low-risk *Listeria monocytogenes* isolates

Analyses for hemolytic property, virulence genes, growth patterns, and the heat resistance test showed that *L. monocytogenes* SMFM 201804 SD 5-3 had the highest risk, while *L. monocytogenes* SMFM 2019-FV43 had the lowest risk. Thus, *L. monocytogenes* SMFM 201804 SD 5-3 and *L. monocytogenes* SMFM 2019-FV43 were defined as a high- and low-risk isolates, respectively. *L. monocytogenes* SMFM 201804 SD 5-3, isolated from smoked duck, showed relatively high growth at 4°C, the highest heat resistance, and relatively slow reduction in vacuum condition. *L. monocytogenes* SMFM 2019-FV43, isolated from the enoki mushrooms, had the lowest growth at 4°C, fast reduction under vacuum conditions, and low heat resistance compared with *L. monocytogenes* SMFM 201804 SD 5-3. For tracing the causes of these different characteristics between high- and low-risk *L. monocytogenes*, WGS was performed.

### 3.8. *De novo* assembly results

The *de novo* assembly results showed that *L. monocytogenes* SMFM 2019-FV43 and *L. monocytogenes* SMFM 201804 SD 5-3 had three contigs. The genome sizes of contigs 1, 2, and 3 for *L. monocytogenes* SMFM 2019-FV43 were 3,071,014, 60,439, and 6,273 bp, respectively, with GC contents of 37.92, 36.69, and 50.39%, respectively. The genome sizes of contigs 1, 2, and 3 for *L. monocytogenes* SMFM 201804 SD 5-3 were 3,038,302, 57,472, and 6,011 bp, with GC contents of 38.05, 36.04, and 50.39%, respectively.

### 3.9. Genetic variations for virulence factors

**Figure 5:**
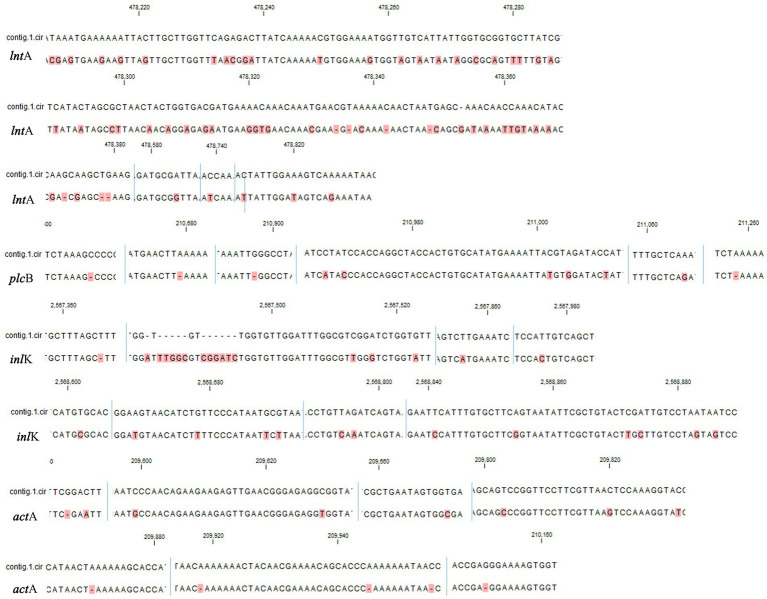
Single nucleotide variants in virulence factors (*lnt*A, *plc*B, *inl*K, and *act*A) of *Listeria monocytogenes* SMFM 2019-FV43 and *Listeria monocytogenes* SMFM 201804 SD 5-3. contig.1.cir: *Listeria monocytogenes* SMFM 2019-FV43 contig 1; blue line: nucleotide sequence boundary. All dissimilar nucleotides are indicated in red.

Single nucleotide variants (SNVs) are variations reflecting differences between a single base [Ministry of Food and Drug Safety (MFDS), 2019]. The gene sequences of the virulence factors of *L. monocytogenes* SMFM 201804 SD 5-3 and *L. monocytogenes* SMFM 2019-FV43 were mapped to identify genetic variations. We identified 45 virulence genes in the two isolates. For these 45 virulence genes, 41 SNVs were found between the isolates (Table 6). The SNVs were found in 91% (41/45) of virulence genes. Among the genes, the four pathogenic genes (*lnt*A, *plc*B, *inl*K, and *act*A), in which the frequency of SNVs was high, were focused (Figure 5). *lnt*A in *L. monocytogenes* encodes a secretory protein that controls the expression of IFN-stimulated genes. This allows the bacterium to govern both the induction and repression of the host cell immune response to optimize conditions for specific stages of infection or colonization (Camejo et al., 2011). *plc*B encodes PC-PLC, which is involved in effective escape from the phagocytic vacuole to the cytoplasm (Gründling et al., 2003; Hadjilouka et al., 2016). Dortet et al. (2011) confirmed *inl*K as a gene highly activated during infection and that it may play a role in the infection process. *actA* is responsible for actin-based motility and the spread of *L. monocytogenes* to neighboring cells (Vàzquez-Boland et al., 2001).

**Table 6:** Single nucleotide variants in virulence genes of *Listeria monocytogenes* SMFM 2019-FV43 and *Listeria monocytogenes* SMFM 201804 SD 5-3.

Virulence genes	SNV frequency % (no.)	Virulence genes	SNV frequency % (no.)
*lntA*	9.78% (62/634)	*vir*R	0.30% (2/677)
*inl*K	1.70% (31/1820)	*lgt*	0.24% (2/832)
*act*A	1.27% (13/1022)	*lap*B	0.23% (12/5138)
*plc*B	1.16% (10/865)	*Oat*A	0.21% (4/1884)
*che*Y	0.84% (3/359)	*iap/cwh*A	0.21% (3/1440)
*bsh*	0.72% (7/978)	*pdg*A	0.21% (3/1399)
*prf*A	0.70% (5/714)	*plc*A	0.21% (2/954)
*inl*J	0.65% (12/1859)	*inl*A	0.17% (4/2402)
*lap*	0.62% (16/2599)	*inl*P	0.17% (2/1167)
*lis*R	0.59% (4/681)	*vir*S	0.17% (1/582)
*hly*	0.57% (9/1584)	*agr*C	0.15% (2/1296)
*inl*F	0.53% (13/2470)	*lis*K	0.14% (2/1452)
*ami*	0.47% (13/2751)	*stp*	0.13% (1/758)
*mpl*	0.46% (7/1530)	*srt*B	0.13% (1/741)
*lpe*A	0.43% (4/933)	*aut*	0.12% (2/1718)
*agr*A	0.41% (3/726)	*hbp*2	0.12% (2/1710)
*dlt*A	0.39% (6/1533)	*opp*A	0.12% (2/1677)
*hpt*	0.36% (5/1384)	*inl*B	0.11% (2/1890)
*fbp*A	0.35% (6/1712)	*prs*A2	0.11% (1/881)
*che*A	0.32% (6/1857)	*lpl*A1	0.10% (1/966)
*vip*	0.32% (4/1255)		

Therefore, these variations of virulence genes may affect the high risk of *L. monocytogenes* SMFM 201804 SD 5-3. In particular, pathogenic genes (*lnt*A, *plc*B, *inl*K, and *act*A) of the strain could improve attachment and invasion of host cells and affect the host cell immune response.

### 3.10. Genetic variations related to growth at 4°C

**Figure 6:**
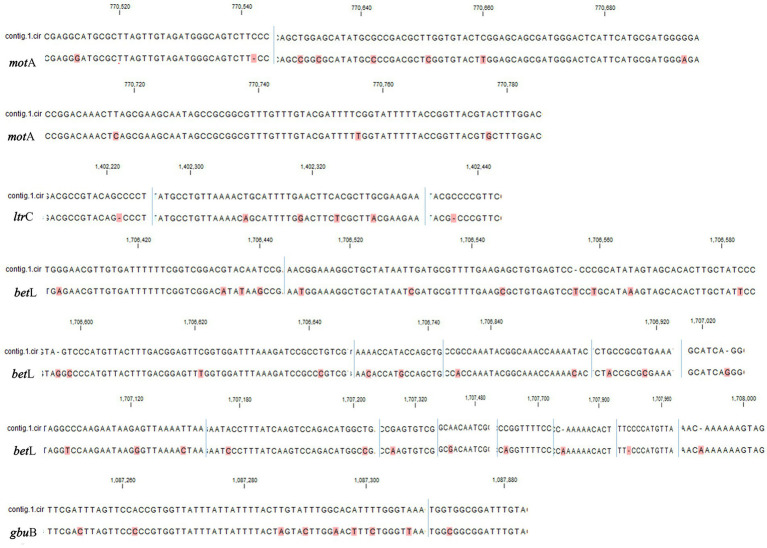
Single nucleotide variants in low-temperature growth related factors of *Listeria monocytogenes* SMFM 2019-FV43 and *Listeria monocytogenes* SMFM 201804 SD 5-3 (*motA*, *ltrC*, *betL*, and *gbuB*). contig.1.cir: *Listeria monocytogenes* SMFM 2019-FV43 contig 1; blue line: nucleotide sequence boundary. All dissimilar nucleotides are indicated in red.

We identified genes related to growth at 4°C for *L. monocytogenes* SMFM 2019-FV43 and *L. monocytogenes* SMFM 201804 SD 5-3 and also found genetic variations in these genes. In all, 26 genes were identified in the two isolates. Among these 26 genes, 18 SNVs were identified (Table 7). The SNVs were found in 69% (18/26) of cold resistance genes. Among the genes, the four pathogenic genes (*motA*, *ltrC*, *betL*, and *gbuB*), in which the frequency of SNVs was high, were focused (Figure 6). Reportedly, *gbu* (*gbu*A, *gbu*B, and *gbu*C) and *bet*L encode proteins involved in the betaine uptake system, and *L. monocytogenes* accumulates betaine when grown at low temperature, which functions as a cryoprotectant and osmoprotectant (Ko et al., 1994; Wemekamp-Kamphuis, 2003). Single and multiple deletions of these genes significantly reduce the viability of *L. monocytogenes* when exposed to low temperatures (Ko and Smith, 1999; Wemekamp-Kamphuis, 2003). *ltr*C is a stress-response gene essential for the growth of *L. monocytogenes* at cold temperatures (e.g., 4°C) (Zheng and Kathariou, 1995; Chan et al., 2007); *flh*A and *mot*A also play a role in the cold tolerance of *L. monocytogenes* (Mattila et al., 2011). In this study*, L. monocytogenes* SMFM 201804 SD 5-3 had a high growth rate at 4°C than *L. monocytogenes* SMFM 2019-FV43 did, with a difference of 1.1 Log CFU/mL after storage for 288 h (Figure 3). Thus, it is reasonable to consider that *L. monocytogenes* SMFM 201804 SD 5-3 can sustain and grow at low temperatures, compared with *L. monocytogenes* SMFM 2019-FV43, given the genetic variations *gbu*B, *bet*L, *mot*A, and *ltr*C (which are related to cold stress) in the former.

**Table 7:** Single nucleotide variants in low-temperature growth-related genes of *Listeria monocytogenes* SMFM 2019-FV43 and *Listeria monocytogenes* SMFM 201804 SD 5-3.

Low-temperature growth-related genes	SNV frequency % (no.)
*bet*L	1.97% (33/1671)
*mot*A	1.30% (11/851)
*ltr*C	1.20% (6/499)
*gbu*B	1.06% (9/849)
*trx*B	0.83% (8/959)
*sig*B	0.77% (6/778)
*gro*EL	0.55% (9/1625)
*csp*B	0.50% (1/201)
*rsb*U	0.50% (1/201)
*ltr*A	0.45% (5/1113)
*ltr*B	0.44% (7/1590)
*fbp*	0.35% (6/1712)
*flh*A	0.24% (5/2074)
*yyc*J	0.24% (2/831)
*clp*P	0.17% (1/596)
*opp*A	0.12% (2/1677)
*gbu*C	0.11% (1/903)
*gbu*A	0.08% (1/1194)

### 3.11. Genetic variations related to heat resistance

We analyzed heat resistance-related genes and genetic variations in *L. monocytogenes* SMFM 2019-FV43 and *L. monocytogenes* SMFM 201804 SD 5-3. We identified 21 genes related to heat resistance. For these 21 genes, 16 SNVs were identified (Table 8). The SNVs were found in 76% (16/21) of heat resistance-related genes. The *cts*R involved in regulating stress and heat shock, may coordinate the expression of stress genes (*clp*C, *clp*P, and *clp*E) in *L. monocytogenes* under stress conditions and in infected hosts. Stress-induced *clp*C, *clp*P, and *clp*E proteins are crucial for virulence (Nair et al., 2000). Regulation of gene expression in response to environmental stress is essential for bacterial survival (Akbar et al., 1997). Stress proteins play an important role in virulence, and therefore, variations in the heat resistance-related genes may be the plausible reasons underlying the heat resistance of *L. monocytogenes* SMFM 201804 SD 5-3.

**Table 8:** Single nucleotide variants in heat resistance-related genes of *Listeria monocytogenes* SMFM 2019-FV43 and *Listeria monocytogenes* SMFM 201804 SD 5-3.

Heat resistance-related gene	SNV frequency % (no.)
*clp*E	0.51% (11/2169)
*lmo*0942	0.37% (3/806)
*gro*EL	0.35% (1/285)
*gro*ES	0.35% (1/285)
*lmo*0963	0.33% (3/914)
*lmo*0231	0.29% (3/1023)
*dna*J	0.27% (3/1131)
*dna*K	0.22% (4/1841)
*lmo*1279	0.21% (3/1410)
*lmo*0292	0.20% (3/1502)
*clp*P	0.17% (1/596)
*grp*E	0.17% (1/576)
*lmo*0038	0.17% (1/572)
*lmo*1138	0.17% (1/572)
*clp*B	0.12% (3/2600)
*hrc*A	0.10% (1/1038)

## 4. Conclusion

*Listeria monocytogenes* isolates have different risks, with different survival responses and pathogenicity under stressful conditions. These differences are caused by SNVs in virulence genes, low-temperature-related genes, and heat resistance-related genes. Our results support the position that *L. monocytogenes* SMFM 201804 SD 5-3 is a high-risk isolate whereas *L. monocytogenes* SMFM 2019-FV43 is a low-risk one.

## Data availability statement

The datasets presented in this study can be found in the NCBI database. The Bioproject IDs are PRJNA958457 and PRJNA958458.

## Author contributions

JR, YC, and YY made significant contributions to the manuscript and agree to its publication. YY and YC conceived and designed the study. JR performed laboratory experiments. All authors analyzed the data, draft the manuscript, and approved the final manuscript.

## Conflict of interest

The authors declare that the research was conducted in the absence of any commercial or financial relationships that could be construed as a potential conflict of interest.

## Publisher’s note

All claims expressed in this article are solely those of the authors and do not necessarily represent those of their affiliated organizations, or those of the publisher, the editors and the reviewers. Any product that may be evaluated in this article, or claim that may be made by its manufacturer, is not guaranteed or endorsed by the publisher.
